# Uncertainty Quantification of First Fix in a Time-Differenced Carrier Phase Observation Model

**DOI:** 10.3390/s25113480

**Published:** 2025-05-31

**Authors:** Hakim Cherfi, Julien Lesouple, Joan Solà, Paul Thevenon

**Affiliations:** 1 Fédération ENAC ISAE-SUPAERO ONERA, Université de Toulouse, 7, Avenue Edouard Belin, 31400 Toulouse, France; julien.lesouple@enac.fr (J.L.); thevenon@recherche.enac.fr (P.T.); 2 Institut de Robòtica i Informàtica Industrial (IRII), CSIC-Universitat Politècnica de Catalunya, 08028 Barcelona, Spain; jsola@iri.upc.edu

**Keywords:** GNSS, TDCP, uncertainty quantification, odometry, positioning error, navigation

## Abstract

This paper presents an uncertainty quantification analysis of the first fix in a time-differenced carrier phase (TDCP) observation model. TDCP is a widely used method in GNSS-based odometry for precise positioning and displacement estimation. A key point in the TDCP modeling is the assumption that the GNSS receiver’s initial position is perfectly known, which is never exactly the case in real-world applications. This study assesses the impact of initial position errors on estimated displacement by formulating a correct TDCP model and a misspecified one, where the first position is not correct. Theoretical derivations provide a generic framework of estimation under the misspecified model and its associated mean squared error (MSE), as well as estimation performance bounds through the misspecified Cramer Rao bound (MCRB) for the considered case. These theoretical considerations are then used to build an estimator of the receiver’s displacement, with comparisons to the MCRB for performance evaluation. Extensive simulations using realistic GNSS geometry assess the influence of a first-fix error under various conditions, including different time intervals, first-fix error norms, and first-fix error direction. As an example, it is shown that for the considered geometry, if a TDCP of $t_{2}-t_{1}=1$ s is built with an initial first fix error norm $∥\Delta r_{1}∥=10\;m$, then it introduces an estimation of the displacement, with an error of norm equal to 1.3 mm, at most. The results indicate that the displacement estimation error is linearly related to the initial position error and the time interval between observations, highlighting the importance of accurate first-fix estimation for reliable TDCP-based odometry. The findings contribute to highlighting the order of magnitude of errors on solutions as a function of the error on parameters.

## 1. Introduction

Odometry is a fundamental technique used in robotics, autonomous vehicles, and geospatial applications for estimating the change in position of a moving system. It relies on sensors or data from systems like wheel encoders, inertial measurement units, vision sensors, lasers, or global navigation satellite systems (GNSS) to measure the motion of a vehicle or object. GNSS-based odometry has become an essential tool for achieving precise and reliable positioning and displacement estimation over long distances.

In GNSS-based odometry, the system estimates its positions and/or displacements by processing satellite observations. One of the most precise observations is the carrier phase observation. The carrier phase refers to the phase of the carrier wave used by satellites to transmit signals. This carrier phase observation is particularly valuable because it provides much higher accuracy (on the order of millimeters) compared to standard pseudorange measurements (with meter-level accuracy), making it ideal for precise positioning and odometry. However, this observation is ambiguous, the ambiguity being the integer number of wavelengths traveled by the signal. One way to get rid of this ambiguity, if constant over time, is to use the subtraction of two consecutive carrier phase observations and form what is known as the time-differenced carrier phase (TDCP) observation. This method is widely used in GNSS-based odometry to estimate the change in position over time. In TDCP, it is assumed that the integer ambiguities present in both carrier phase observations cancel out when the subtraction is built, allowing for highly accurate displacement estimation.

Uncertainty quantification refers to the process of quantifying the uncertainties in a quantity of interest by propagating the uncertainties in the input of a system. Uncertainty in GNSS measurements arises from various factors, such as observation noise or an approximate assumed position that is used when building observation models. Reviews about sources of error and their impact within GNSS systems are available in [1].

This paper proposes to focus on the TDCP model: when constructing the TDCP equations as a function of the receiver displacement, the receiver’s position must be known at one of the two epochs. This knowledge on position is never perfect and can affect the estimated displacement. To account for this, uncertainty quantification is used to assess the impact of this inaccuracy on the displacement estimate. By quantifying the uncertainty in the receiver’s position, one can evaluate how this error propagates through the TDCP equations and influences the displacement estimation. This helps in understanding the potential error margins and ensuring that the odometry results remain reliable, even when position uncertainties are present, thus improving the reliability of GNSS-based odometry in real-world applications.

## 2. Related Work

### 2.1. TDCP Models

Using a GNSS receiver, a system could estimate its speed vector at a given instant by means of Doppler observations. Once integrated, these speeds provide an estimation of the system’s displacement. It is known that the Doppler observations, when integrated, are noisier than carrier phase observations, though not sensitive to cycle slip [2]. For this reason, it is interesting to estimate a receiver displacement between two instants by means of TDCP.

In [3], the authors assume a perfectly known receiver position at the first epoch and use this information to run a Kalman filter with TDCP and substracted TDCP observations between satellites, along with a cycle slip detector. The source of the first position is not specified in the experiments. Also, the receiver-to-satellite unit vectors at the two epochs are known. To assess potential cycle slips, the cycle slip detector first generates a set of valid solutions (from a previous estimate, integrated IMU measurements and IMU specifications), and checks if the TDCP solution is within that set.

In [3,4], the authors build substracted TDCPs, i.e., the subtraction of TDCPs between two different satellites, in order to remove the receiver clock bias difference. The subtraction is done with the satellite with the highest elevation, as the receiver most likely receives a direct signal from this one. Still, to estimate the receiver displacement with these subtracted TDCPs, the authors assume known receiver-to-satellite vectors and receiver position at the first epoch of substracted TDCPs.

Some authors go further in the approximations of the TDCP model. In [5,6], the receiver-to-satellite unit vectors are assumed constant at the two TDCP epochs, leading to a unique receiver-to-satellite vector. It should be noted that this approximation requires the knowledge of the receiver position. Then, the receiver and satellite displacement vectors are projected onto this vector thanks to a scalar product. This makes the TDCP a linear model of the receiver displacement.

The same approximation is made in [2,7], where the author explains that the TDCP model is linearized around the estimated position at the previous epoch. Also, the authors build the single difference of carrier phase observations between satellites and between epochs.

In [8], a similar TDCP model is built, with a time between the two epochs ranging from 1 s to 60 s. Regarding the geometric part, the model includes two terms, the first one being a change of range between the satellite and the receiver, which requires the receiver position, and a second term where the estimated receiver displacement vector is projected onto the unit line-of-sight vector at the second epoch, which also requires the receiver position to be known. Also, a single line-of-sight vector is considered, even if the time of interval is 60 s. This TDCP model is used to build a TDCP factor inside a factor graph optimization problem. At the first epoch, a first position is set from a prior factor, and for other epochs, the first position is set from previous nodes. It should be noted that cycle slip factors are used to handle cycle slips.

In [9], the geometric part of the TDCP model is split into three terms, the first one due to the satellite motion along the line-of-sight, a second term that contains the receiver position at the first epoch, and the last term is a linear term of the receiver displacement and of the line-of-sight vector at the second epoch. All these terms require the knowledge of the receiver coordinates at the two epochs.

In [10], the authors leave the TDCP model as a function of the two receiver positions. The TDCPs are used in a factor graph optimization problem, where relative displacement factors from preintegrated inertial measurements are used. In the experimental part of their work, their proposed method outperforms two filtering methods that also use TDCP observations. This is likely due to a prior factor that sets the receiver position at the first epoch, although no details are given regarding this assumption.

Overall, within the GNSS community, many approximations of the TDCP model are provided, which all assume the known receiver coordinates in order to estimate a displacement.

### 2.2. Uncertainty Quantification

Uncertainty quantification can be defined as the process of assessing (and if possible, reducing) the impact of an uncertainty within a system on its output. It is a very general topic that applies to any engineering problem. In the case of a deterministic system, the uncertainty can either come from a lack of knowledge or from a probabilistic modeling of it. Indeed, when the system is probabilistic, the uncertainty directly comes from it. Regarding the quantification, multiple methods exist, depending on where the uncertainty comes from.

In [11], the authors deliver a Python library for uncertainty quantification, for very general cases, ranging from physics to applied mathematics, and with many algorithms.

Within the GNSS community, uncertainty quantification has already been applied. For example, in [12], the authors consider multiple sources of uncertainty, which propagates in GNSS reflectometry observations to estimate sea level. The quantification is made using the RMSE metric. In [13], the authors estimate a GNSS receiver position using noisy observations. The uncertainty is modeled by an Ornstein–Uhlenbeck random process, a Brownian random process, with learned parameters. The observations are run in a Kalman filter, and they find out that all these methods outperform, in terms of uncertainty, the direct approach which consists of running the Kalman Filter in a traditional way. In [14], from confidence volumes of Gaussian distributions, the authors provide analytical extensions of these volumes into zonotopes that take into account uncertainties of an observation model containing potential biases and/or random errors.

### 2.3. Overview of Proposed Method

In the estimation community, work on estimators built with misspecified models exist [15,16,17]. On the other hand, in the GNSS community, to the best of the author’s knowledge, the impact of a misspecified first position in a TDCP model has never been studied. Therefore, a correct TDCP model, as well as a misspecified one, is going to be built. The misspecified one will be different from the correct one by the fact that the true receiver position at the first epoch is never known perfectly. The receiver displacement will be estimated, and the receiver position at the second epoch will be deduced from the receiver position at the first epoch and the estimated displacement. No approximations will be made in these models. They will contain the subtraction of ranges, but these ranges will not be projected onto a line-of-sight vector, as has been done by previously mentioned authors. These two models will be compared experimentally, with multiple values around the true receiver position at the first epoch, in order to assess the importance of the receiver position at the first epoch in a TDCP model to properly estimate a receiver displacement.

## 3. Carrier Phase Observation Model and TDCP

### 3.1. Carrier Phase Observation Model

A GNSS receiver at epoch *t* of coordinates $r_{t}\in R^{3}$ with a clock bias with respect to GPS time $dt_{t}^{r}$ is tracking a satellite of coordinates $s_{t}\in R^{3}$ with a clock bias with respect to GPS time $dt_{t}^{s}$.

The carrier phase observation is modeled as follows:

(1) $$\lambda \Phi_{t}=∥s_{t}-r_{t}∥+c\;\cdot \;\left(dt_{t}^{r}-dt_{t}^{s}\right)+\lambda N_{t}-I_{t}+T_{t}+m_{t}+\varepsilon_{t}^{\Phi }$$

where the subscript *t* is used to indicate that the parameters are expressed at epoch *t*, $\Phi_{t}$ is the carrier phase raw measurement, λ is the wavelength of the considered frequency band, *c* is the speed of light, $I_{t}$ is the ionospheric delay, $T_{t}$ is the tropospheric delay, $m_{t}$ is the multipath error, $N_{t}\in Z$ is the integer ambiguity, $\varepsilon_{t}^{\Phi }$ is a centered Gaussian noise with standard deviation $\sigma_{\phi }$, and the norm of a vector $v\in R^{3}$ is defined as $∥v∥=\sqrt{v^{\;T}v}$.

In this model, the satellite coordinates $s_{t}$ and clock bias $dt_{t}^{s}$ are known from the ephemeride information. In a precise point positioning (PPP) framework, the unknown quantities that are estimated are the receiver coordinates $r_{t}$, the clock bias $dt_{t}^{r}$, the integer ambiguity $N_{t}$, and possible other atmospheric states.

### 3.2. TDCP Observation Model and Corrections

Considering two carrier phase observations as in Equation (1) at epochs $t_{1}$ and $t_{2}$ from the same satellite, the TDCP model is built:

(2) $$\begin{array}{cc} \lambda (\Phi_{2}-\Phi_{1}) & =∥s_{2}-r_{2}∥-∥s_{1}-r_{1}∥+c\;\cdot \;\left(dt_{2}^{r}-dt_{2}^{s}\right)-c\;\cdot \;\left(dt_{1}^{r}-dt_{1}^{s}\right)\\ \; & +\lambda \;\cdot \;\left(N_{2}-N_{1}\right)-I_{2}+I_{1}+T_{2}-T_{1}+m_{2}-m_{1}+\varepsilon_{2}^{\Phi }-\varepsilon_{1}^{\Phi }. \end{array}$$

Correcting all the atmospheric terms [18,19] and satellite clock biases, assuming the difference between the two multipath terms is an additive centered Gaussian noise, and assuming no cycle slip ($N_{2}-N_{1}=0$), the model (2) becomes:

(3) $$\begin{array}{cc} \lambda ({\tilde{\Phi }}_{2}-{\tilde{\Phi }}_{1}) & =∥s_{2}-r_{2}∥-∥s_{1}-r_{1}∥+c\;\cdot \;\left(dt_{2}^{r}-dt_{1}^{r}\right)+\varepsilon \end{array}$$

where ${\tilde{\Phi }}_{t}$ corresponds to the corrected carrier phase observation, ε is the contribution of the two independent errors $\varepsilon_{2}^{\Phi }-\varepsilon_{1}^{\Phi }$, as well as all other mutually independent Gaussian centered residual errors after correction. Therefore, ε is a centered Gaussian noise with standard deviation $\sigma_{\mathrm{TDCP}}>\sqrt{2}\sigma_{\phi }$. The assumption of constant ambiguity requires the use of a properly working cycle slip detector [20].

Written this way, the model in Equation (3) is a function of the receiver coordinates $r_{1}$ and $r_{2}$ and receiver clock biases $dt_{1}^{r}$ and $dt_{2}^{r}$. While the use of multiple observations from other satellites (at least 4 for each epoch) with this model would still permit one to estimate all of these quantities, depending on the time elapsed between epochs $t_{1}$ and $t_{2}$, the obtained result would be highly sensitive to noise, and the mean error of an optimal unbiased estimator would be very large. This is due to the fact that between two epochs, the receiver-to-satellite unit vectors would not change much, hence a linear approximation that is badly conditioned. This point discourages a user from estimating all these quantities directly using TDCP observations unless considering a very large time interval between the two considered epochs.

Regarding the ephemerides, in [9], the authors compared the use of final products (i.e., high-precision satellite coordinates in post-processing conditions) versus real-time navigation data and figured out that the use of final products would not improve the solution with respect to the use of navigation data, as between the two epochs, if the same set of ephemerides is used, the satellites positions errors are approximately constant. On the other hand, they figured out that when the set of ephemeride parameters is updated, a gap appears in the satellite positions, which adds an important error in a TDCP model if this update happens between the two epochs. To cancel this phenomenon, it is therefore important to use the same set of ephemerides in a TDCP model.

### 3.3. TDCP Model with True Receiver Position and Receiver Displacement

The model in Equation (3) can be expressed in terms of receiver displacement and clock bias difference. The receiver displacement vector between epochs $t_{1}$ and $t_{2}$ is defined as $r_{1,2}≜r_{2}-r_{1}$, and the receiver clock bias difference between epochs $t_{1}$ and $t_{2}$ is defined as $dt_{1,2}^{r}≜dt_{t_{2}}^{r}-dt_{t_{1}}^{r}$. With these notations, the model in Equation (3) is written as:

(4) $$\lambda \left({\tilde{\Phi }}_{2}-{\tilde{\Phi }}_{1}\right)=f_{r_{1}}\left(r_{1,2},dt_{1,2}^{r}\right)+\varepsilon .$$

where

(5) $$\begin{array}{cc} f_{r_{1}}:R^{3}\times R & \to R\\ \left(x_{1},x_{2}\right) & \mapsto ∥s_{2}-\left(r_{1}+x_{1}\right)∥-∥s_{1}-r_{1}∥+c\;\cdot \;x_{2}. \end{array}$$

The index indicates that this function is not a function of the receiver coordinates but of its displacement, with the position at epoch $t_{1}$ set to $r_{1}$, and not of a receiver clock bias, but of its clock bias difference. Given a set of visible satellites, this observation model and TDCP observations can be built for each satellite. The set of models and observations can then be used to estimate the receiver displacement vector and clock bias difference by solving the maximum likelihood problem. In practice, the receiver position $r_{1}$ is not perfectly known. Therefore, in the next subsection, an error term will be introduced, leading to a different model.

### 3.4. TDCP Model with Assumed Receiver Position and Receiver Displacement

The previous model $f_{r_{1}}$ in Equation (5) assumed perfect knowledge of the true receiver coordinates $r_{1}$. In practice, these coordinates can be inaccurate. A first fix denotes an estimated initial position ${\hat{r}}_{1}$ of the true initial position $r_{1}$. Therefore, the error between the true coordinates and the assumed ones are defined as $\Delta r_{1}≜{\hat{r}}_{1}-r_{1}$. From these notations, the true model is the one in Equation (5) while the assumed one is:

(6) $$\begin{array}{cc} f_{{\hat{r}}_{1}}:R^{3}\times R & \to R\\ \left(x_{1},x_{2}\right) & \mapsto ∥s_{2}-\left({\hat{r}}_{1}+x_{1}\right)∥-∥s_{1}-{\hat{r}}_{1}∥+c.x_{2}. \end{array}$$

One can notice that ${\hat{r}}_{1}=r_{1}\Leftrightarrow f_{{\hat{r}}_{1}}=f_{r_{1}}$.

In order to quantify the difference between the two models as a function of $\Delta r_{1}$, one defines the difference between the assumed model Equation (6) and the true model Equation (5), expressed as a function of the error $\Delta r_{1}$, for the true receiver displacement and the true receiver clock bias difference:

(7) $$\begin{array}{cc} \Delta f:R^{3} & \to R\\ \Delta r_{1} & \mapsto f_{r_{1}+\Delta r_{1}}\left(r_{1,2},dt_{1,2}^{r}\right)-f_{r_{1}}\left(r_{1,2},dt_{1,2}^{r}\right). \end{array}$$

It can be noted that this application cancels all the clock biases terms, and only the difference in the geometric ranges remains:

(8) $$\begin{array}{cc} \Delta f(\Delta r_{1}) & =∥s_{2}-\left(r_{1}+\Delta r_{1}+r_{1,2}\right)∥-∥s_{1}-\left(r_{1}+\Delta r_{1}\right)∥\\ \; & -∥s_{2}-\left(r_{1}+r_{1,2}\right)∥+∥s_{1}-r_{1}∥. \end{array}$$

Therefore, the first fix ${\hat{r}}_{1}$ may have an impact on the estimated receiver displacement and clock bias difference.

In general, the use of a misspecified model to estimate parameters has an impact on the estimation. The objective of the following sections is to experimentally assess the estimation error due to a fix error $\Delta r_{1}$.

### 3.5. Estimation of a Receiver Displacement and Clock Bias Difference from TDCP Observations

**Figure 1:**
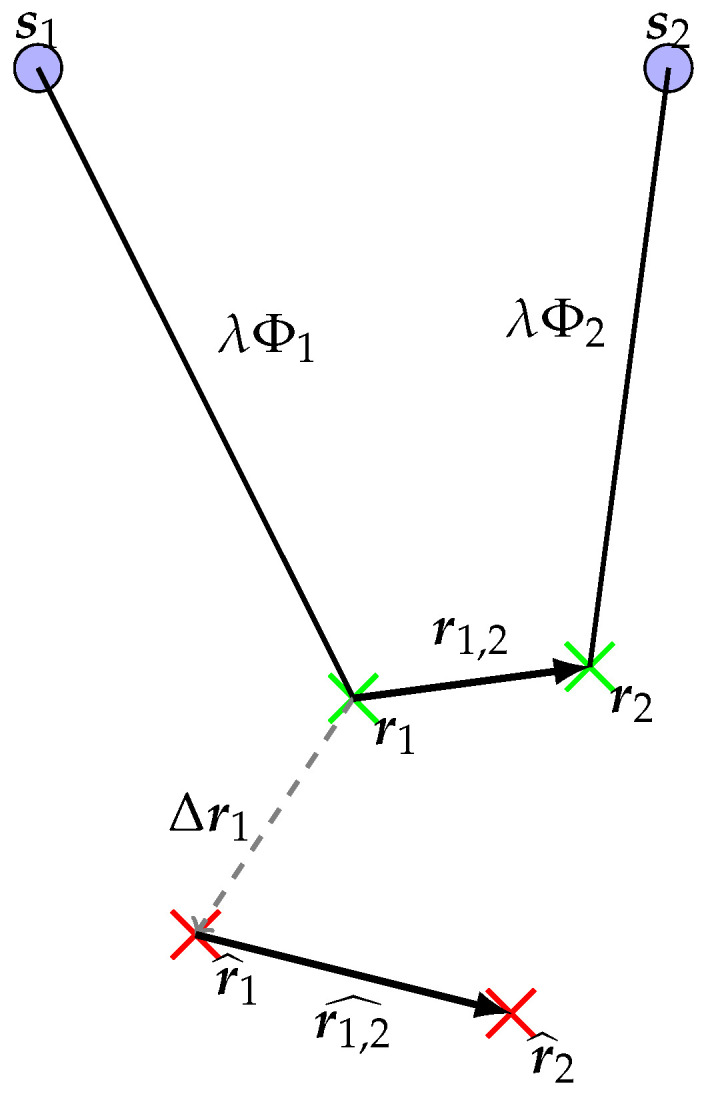
Representation of the TDCP model in 2D. The blue disks represent the satellite positions at the two epochs, the green crosses represent the true receiver positions, the red ones represent the assumed receiver positions.

Now that a misspecified TDCP model has been written in Equation (6), one can estimate a receiver displacement and clock bias difference from a set of *m* visible satellites at the two epochs $t_{1}$ and $t_{2}$ and from the set of TDCP observations at these epochs. A receiver’s displacement estimator is going to be defined and is denoted $\hat{r_{1,2}}$. The second epoch receiver’s position estimator is denoted ${\hat{r}}_{2}≜{\hat{r}}_{1}+\hat{r_{1,2}}$. A 2D representation of the TDCP model, for a single satellite, is visible in Figure 1.

To assess the degradation of the displacement estimation due to a misspecified first fix, one can explicitly write this estimator, compute its statistical properties, and assess the impact of $\Delta r_{1}$ on it. In the next section, theoretical elements are provided to achieve these objectives.

## 4. Estimation: Computation of Mean Squared Error and Its Lower Bound

This section uses a more general framework than the one used so far in this article and provides the theoretical elements to build an estimator of a vector of parameters living in a Euclidean space from a multivariate Gaussian random variable. In the considered framework, the mean value of the Gaussian random variable is misspecified. This uncertainty is going to impact the estimator’s performances, and one proposes to quantify this impact. Additionally, this section provides a theory to compute the statistical properties of this estimator. Finally, the link with the original problem (the estimation of a GNSS receiver’s displacement and clock bias difference, from a set of TDCP observations) is made.

### 4.1. Estimator of a Real Vector

Given an unknown vector $\theta \in R^{n}$ and a function $g:R^{n}\to R^{m}$ differentiable at θ, one obtains a vector of observations:

(9) y=g(θ)+ε,ε∼N(0,R).

where $N\left(\mu ,\Sigma \right)$ denotes the multivariate Gaussian distribution with mean vector μ and covariance matrix Σ. In this study, the function *g* is unknown, i.e., the construction of an estimator of θ will be based on a function $h:R^{n}\to R^{m}$.

Since y∼N(g(θ),R), but *g* is unknown, one defines the misspecified likelihood function:

(10) $$\begin{array}{cc} q_{y}:R^{n} & \to R\\ x & \mapsto \frac{1}{{\left(2\pi \right)}^{\frac{m}{2}}\sqrt{\left|R\right|}}\exp\left(-\frac{1}{2}{∥y-h\left(x\right)∥}_{R}^{2}\right) \end{array}$$

where ${∥v∥}_{R}^{2}≜v^{\;T}R^{-1}v$. The mismatched maximum likelihood estimator (MMLE) is defined as:

(11) $$\begin{array}{c} \hat{\theta }≜\underset{x\in R^{n}}{\arg\max\;}q_{y}\left(x\right)=\underset{x\in R^{n}}{\arg\min\;}{∥y-h\left(x\right)∥}_{R}^{2} \end{array}$$

as long as the above equation is a measurable function of y. $\hat{\theta }$ is Gaussian if and only if *h* is a linear or affine function. In general, computing its distribution or statistical properties is not straightforward. Instead, an approximation of $\hat{\theta }$ is defined: assuming that the function *h* is differentiable on a set $S\subseteq R^{n}$, then one can choose a vector $\theta_{0}\in S$, and the function can be written:

(12) $$\begin{array}{c} \forall x\in R^{n},h\left(x\right)=h\left(\theta_{0}\right)+H_{\theta_{0}}\left(x-\theta_{0}\right)+o\left(∥x-\theta_{0}∥\right) \end{array}$$

where $H_{\theta_{0}}$ represents the Jacobian matrix of *h* at $\theta_{0}$. This way, the function *h* is replaced by its first order development at $\theta_{0}$ in the misspecified likelihood Equation (10), leading to:

(13) $$\begin{array}{c} \hat{\theta }\approx \underset{x\in R^{n}}{\arg\min\;}{∥y-\left(h\left(\theta_{0}\right)+H_{\theta_{0}}\left(x-\theta_{0}\right)\right)∥}_{R}^{2}. \end{array}$$

The vector $\theta_{0}$ can be obtained by running the Gauss–Newton algorithm with a sample of y and an arbitrary initial estimate. Under certain conditions on the function *h* and on the step size [21], the Gauss–Newton algorithm is guaranteed to converge to a local minima and give an estimate (denoted $\theta_{0}$). The proposed approach assumes that the function *h* is not too nonlinear (in particular, that from the definition of an operator norm, $\left|\;\left|\;\left|H_{\theta_{0}}-H_{\theta }\right|\;\right|\;\right|\approx 0$).

If $H_{\theta_{0}}$ represents an injective application, one defines the matrix:

(14) $$\begin{array}{c} S≜{\left(H_{\theta_{0}}^{\;T}R^{-1}H_{\theta_{0}}\right)}^{-1}H_{\theta_{0}}^{\;T}R^{-1}. \end{array}$$

The solution of the optimization problem Equation (13) is given by:

(15) $$\hat{\theta }\approx S\left(y-h(\theta_{0})\right)+\theta_{0}.$$

In this case, $\hat{\theta }$ is approximated by a Gaussian random vector.

### 4.2. Properties of the Estimator

Now that an estimator Equation (15) is available, some of its statistical properties are provided. One determines that:

- Since y∼N(g(θ),R), the estimators’s mean vector is $E\left[\hat{\theta }\right]=S\left(g\left(\theta \right)-h\left(\theta_{0}\right)\right)+\theta_{0}$;
- The estimator’s bias is $b\left[\hat{\theta }\right]=S\left(g\left(\theta \right)-h\left(\theta_{0}\right)\right)+\theta_{0}-\theta$;
- Since $\hat{\theta }-E\left[\hat{\theta }\right]=S\varepsilon$, the estimator’s covariance matrix is $V\left[\hat{\theta }\right]=SRS^{\;T}={\left(H_{\theta_{0}}^{\;T}R^{-1}H_{\theta_{0}}\right)}^{-1}$.

Defining the mean squared error operator $\mathrm{MSE}\left[\hat{\theta }\right]≜E\left[\left(\hat{\theta }-\theta \right){\left(\hat{\theta }-\theta \right)}^{\;T}\right]$, it can be shown that:

(16) $$\begin{array}{c} \mathrm{MSE}\left[\hat{\theta }\right]=V\left[\hat{\theta }\right]+b\left[\hat{\theta }\right]b{\left[\hat{\theta }\right]}^{\;T}. \end{array}$$

### 4.3. Lower Bound of the Mean Squared Error of a Biased Estimator

Formally, an estimator is a measurable function that takes as input a random variable (often called observations), and with an output space that contains the vector of parameters to estimate. In the estimation theory, it is known that among all estimators, with the same input random variable and the same output space, there exists a lower bound for the mean squared error [22]. Therefore, it is interesting to construct it and compare it with the mean squared error of the considered estimator.

The misspecified Cramer–Rao Bound (MCRB) is defined as [23]:

(17) $$\mathrm{MCRB}≜\left(I_{n}+\Psi_{\theta }\right)I_{\theta }^{-1}{\left(I_{n}+\Psi_{\theta }\right)}^{\;T}+b\left[\hat{\theta }\right]b{\left[\hat{\theta }\right]}^{\;T}$$

where $I_{n}$ represents the identity matrix of size *n*.

The term $\Psi_{\theta }$ represents the Jacobian matrix of the estimator’s bias, denoted $b\left[\hat{\theta }\right]$, at the vector θ, when it is seen as a function of the true vector of parameters θ. The previous section has shown that, in general, the considered estimator $\hat{\theta }$ is biased. Therefore, one defines this bias function as:

(18) $$\begin{array}{cc} \psi :R^{n} & \to R^{n}\\ x & \mapsto S\left(g\left(x\right)-h\left(\theta_{0}\right)\right)+\theta_{0}-x. \end{array}$$

The Jacobian matrix of ψ at θ is given by:

(19) $$\begin{array}{c} \Psi_{\theta }=SG_{\theta }-I_{n} \end{array}$$

where $G_{\theta }$ indicates the Jacobian matrix of *g* at θ.

The term $I_{\theta }$ denotes the Fisher Information matrix [23]. Since y∼N(g(θ),R), the likelihood function is given by:

(20) $$\begin{array}{cc} l_{y}:R^{n} & \to R\\ x & \mapsto \frac{1}{{\left(2\pi \right)}^{\frac{m}{2}}\sqrt{\left|R\right|}}\exp\left(-\frac{1}{2}{∥y-g\left(x\right)∥}_{R}^{2}\right). \end{array}$$

Under some regularity conditions [22], the Fisher Information matrix is defined as:

(21) $$\begin{array}{c} I_{\theta }≜-E\left[d_{\theta }^{2}\left(\log\circ l_{y}\right)\right] \end{array}$$

where $d_{\theta }^{2}f$ corresponds to the Hessian matrix of a function *f* at θ. In the considered case, one can show that:

(22) $$I_{\theta }=G_{\theta }^{\;T}R^{-1}G_{\theta }.$$

Finally, assuming that $G_{\theta }$ represents an injective application, then the Fisher Information matrix invert is well defined.

Finally, a theorem states that [23]:

(23) $$\mathrm{MSE}\left[\hat{\theta }\right]\geq \mathrm{MCRB}$$

where the inequality symbol ≥ represents the Loewner partial order.

### 4.4. Application to the Problem

Now that a theoretical framework has been written, it can be used to estimate a GNSS receiver displacement and clock bias difference from a misspecified TDCP observation model and quantify the impact of this misspecified model on the estimated displacement. The link between the different quantities introduced in the previous sections is summarized in Table 1. In the next section, the receiver displacement and clock bias difference will be estimated from a set of TDCP observations of *m* satellites.

## 5. Experiments

**Figure 2:**
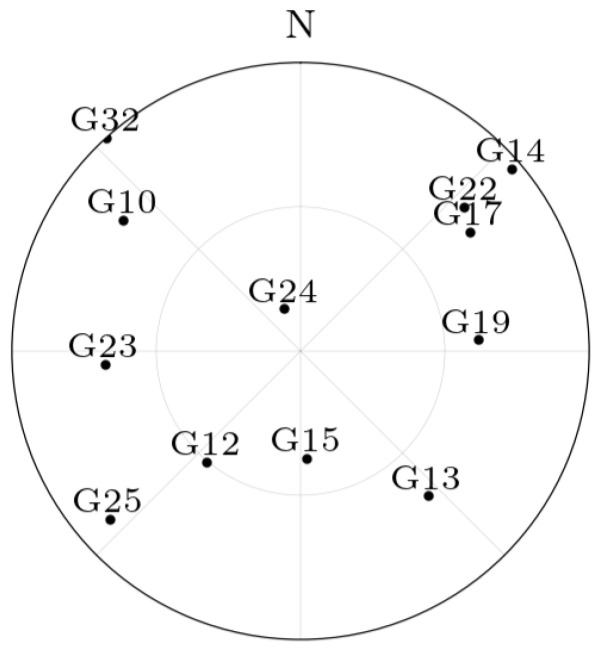
Skyplot of GPS satellites in Toulouse, France, on the first of January 2024, 12:00 UTC.

In this experiment, the set of all visible GPS satellites in Toulouse, France, on the first of January 2024, 12:00 UTC are considered. A skyplot is provided in Figure 2. A synthetic GNSS receiver is constructed, with known coordinates $r_{1}$. Its displacement vector is a sample of a random zero-mean Gaussian random variable with a covariance matrix proportional to the time interval between successive epochs, i.e., $r_{1,2}$ is a sample of the random variable $N\left(0_{3},\left(t_{2}-t_{1}\right)I_{3}\right)$, as this stochastic model captures small random movements of the receiver between epochs while maintaining a realistic representation of a pedestrian receiver over short timescales [24,25]. The receiver clock bias difference $dt_{1,2}^{r}$ is a sample of the law $N\left(0,t_{2}-t_{1}\right)$.

The satellite positions are derived from precise orbit data provided in the Standard Product 3 (SP3) format. These SP3 files offer highly accurate satellite ephemerides, providing the real positions of GPS satellites. This ensures that the data reflect actual satellite orbits, further enhancing the realism of the simulation.

### 5.1. Experimental Uncertainty Quantification of TDCP Model

The first experiment characterizes the TDCP model Equation (7), for a single satellite. A satellite is chosen (G17). For each value of $\Delta r_{1}$, a value for Δf Equation (7) is obtained, indicating the discrepancy between the two TDCP models.

**Figure 3:**
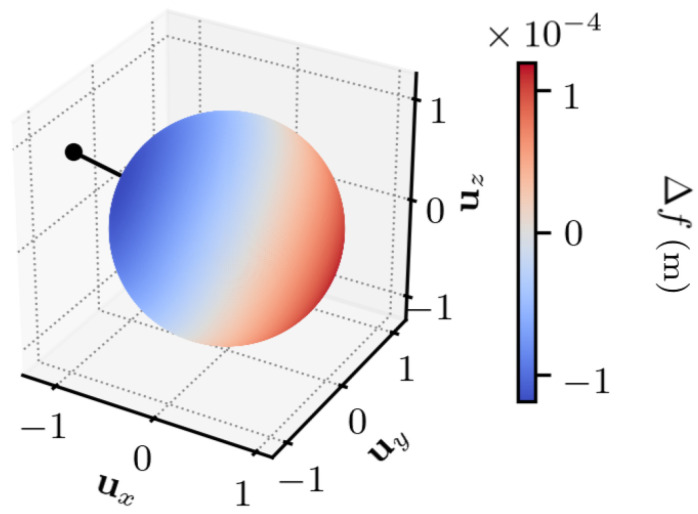
$∥\Delta r_{1}∥\;=1\;m$, $t_{2}-t_{1}=1\;s$. $u_{x}$, $u_{y}$, and $u_{z}$ indicate the *x*, *y*, and *z* components, in the ECEF frame, of $\Delta r_{1}$. The dot indicates the satellite displacement direction.

**Figure 4:**
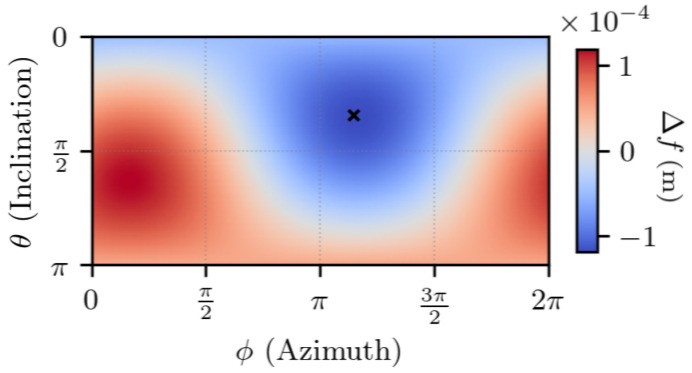
$∥\Delta r_{1}∥\;=1\;m$, $t_{2}-t_{1}=1\;s$. θ and ϕ are the spherical coordinates in the ECEF frame of $\Delta r_{1}$. The cross indicates the satellite displacement direction.

In Figure 3 and Figure 4, the set of values taken by $\Delta r_{1}$ is set to the unit sphere in order to study the importance of its direction. The time between the two epochs is set to $t_{2}-t_{1}=1\;s$, which is a common value in standard receivers. The figures indicate that the discrepancy between the two models is maximum and minimum on two antipodal points, is null on the equator defined by these two antipodal points, and is continuous, as there is no rapid change of value.

In Appendix A, one shows that the maximum and minimum errors are along the direction given by the vector $s_{2}-s_{1}-r_{1,2}$, which approximately corresponds to the satellite displacement, and the error is null for orthogonal vectors to this direction.

Overall, the error is low compared to the norm of the first position error, with a maximum error that is approximately $10^{4}$ lower than $∥\Delta r_{1}∥$.

**Figure 5:**
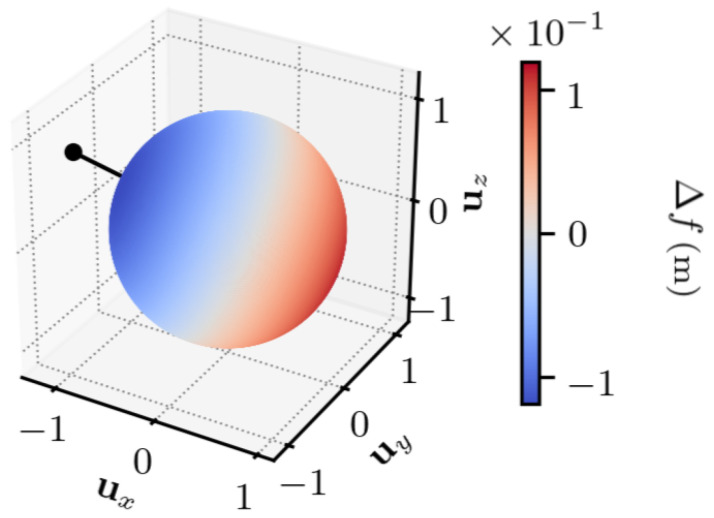
$∥\Delta r_{1}∥\;=1\;km$, $t_{2}-t_{1}=1\;s$. $u_{x}$, $u_{y}$, and $u_{z}$ indicate the *x*, *y*, and *z* components, in the ECEF frame, of $\frac{\Delta r_{1}}{∥\Delta r_{1}∥}$. The dot indicates the satellite displacement direction.

**Figure 6:**
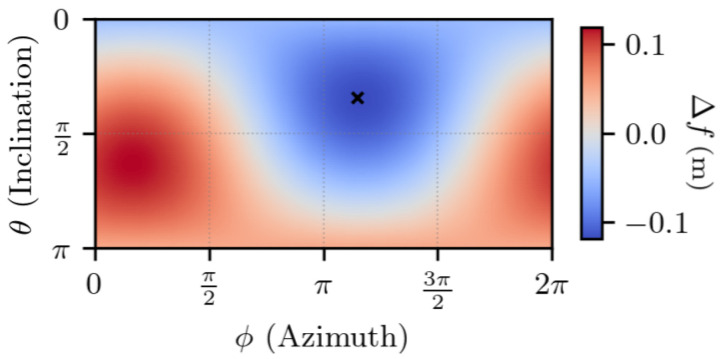
$∥\Delta r_{1}∥\;=1\;km$, $t_{2}-t_{1}=1\;s$. θ and ϕ are the spherical coordinates in the ECEF frame of $\Delta r_{1}$. The cross indicates the satellite displacement direction.

The same experiment is conducted with a much higher first fix error (i.e., a larger sphere). The results are visible in Figure 5 and Figure 6. Again, the worst direction is given by the vector $s_{2}-s_{1}-r_{1,2}$, and one observes a ratio between $∥\Delta r_{1}∥$ and Δf approximately equal to $10^{4}$ in the worst cases.

**Figure 7:**
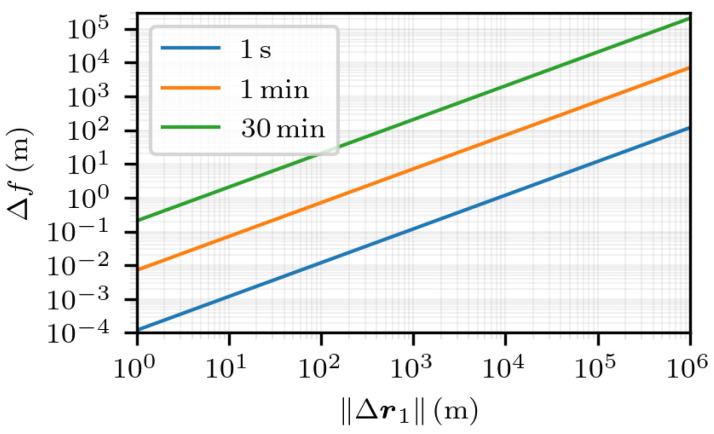
$\Delta r_{1}\propto s_{2}-s_{1}-r_{1,2}$, which corresponds to the worst direction.

The study of the worst case direction is further extended, this time by observing the values taken by Δf on the set $\Delta r_{1}\propto s_{2}-s_{1}-r_{1,2}$, for multiple time intervals $t_{2}-t_{1}$. The results are visible in Figure 7. For convenience, a log-log plot is used. For all the considered time intervals $t_{2}-t_{1}$, the plots indicate a linear relation between the model mismatch Δf and the norm of the first-fix error $∥\Delta r_{1}∥$ since the slope of all the curves is equal to 1 and from Equation (8), Δf(0)=0. For each value of the time interval, the linear coefficient can be read at $∥\Delta r_{1}∥=1\;m$. For example, in the case of $t_{2}-t_{1}=1\;s$, this coefficient is equal to $1.2\times 10^{-4}$. See Appendix B for a computation of these values. It is visible that the linear coefficient depends on the time interval: the greater the time interval, the greater the coefficient. One can conclude that for a given value of $∥\Delta r_{1}∥$, the greater the time interval, the greater the model mismatch.

Finally, the largest value taken by Δf is computed, for the product of a set of values of $∥\Delta r_{1}∥$ and of a set of time intervals between the two observation epochs. This is done in order to explore how the movement of satellites over time would affect the model mismatch. By extending the time between successive observations, the GNSS satellites in the sky change their positions more significantly due to their dynamics. The purpose of this application is to capture a wider range of applications, which can influence the accuracy and reliability of the positioning solutions. These values are visible in Table 2. Omitting rounding errors, the values taken show a bilinear relation with $∥\Delta r_{1}∥$ and $t_{2}-t_{1}$. This result is hard to predict, as Δf is not a linear function of $\Delta r_{1}$, and is even harder to predict for $t_{2}-t_{1}$, as the time interval does not explicitly appear in Δf.

Now that the model mismatch has been characterized for a single satellite, multiple satellites are going to be used to estimate the receiver’s displacement and clock bias difference.

### 5.2. Experimental Uncertainty Quantification of Estimated Displacement with Noiseless Measurements

In this experiment, the receiver’s true positions $r_{1}$ and $r_{2}$ are set as previously explained, and the receiver’s clock bias difference follows a zero-mean Gaussian random variable, with a variance proportional to the time interval $t_{2}-t_{1}$. The set of all visible GPS satellites at the two epochs is used to generate noiseless synthetic TDCP measurements, as in Equation (3), with $\sigma_{\mathrm{TDCP}}=0$. They are used to estimate the receiver displacement and clock bias difference between the two epochs, following Equation (6). The choice regarding noiseless measurements is made in order to have an error of displacement estimation due to a first-fix error only.

For the metric, since the interest is in the estimation of the receiver displacement vector $\hat{r_{1,2}}$, the mean squared error matrix is expressed in a basis such that the submatrix corresponding to the geometrical part is extracted and is denoted MSE, and the square root of the trace of this matrix is computed, i.e., $\sqrt{\mathrm{Tr}\left(\mathrm{MSE}\right)}$. This choice is made in order to plot the mean Euclidean distance error. Also, denoting PDOP the position dilution of precision, this metric is equal to $\sigma_{\mathrm{TDCP}}\times \mathrm{PDOP}$, in the case of independent and identically distributed noise [26].

**Figure 8:**
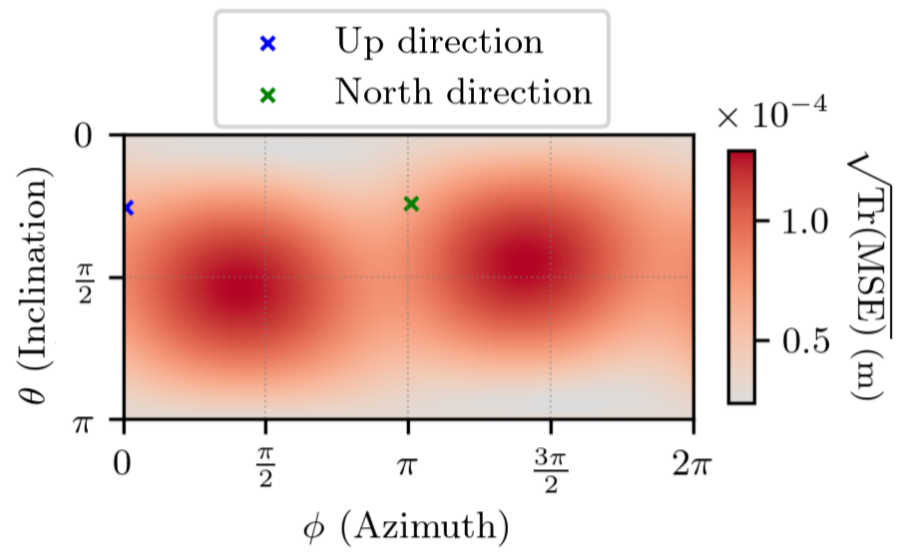
$\sqrt{\mathrm{Tr}\left(\mathrm{MSE}\right)}$ for noiseless measurements, with $∥\Delta r_{1}∥\;=1\;m$, $t_{2}-t_{1}=1\;s$. θ and ϕ are the spherical coordinates, in the ECEF frame, of $\Delta r_{1}$.

**Figure 9:**
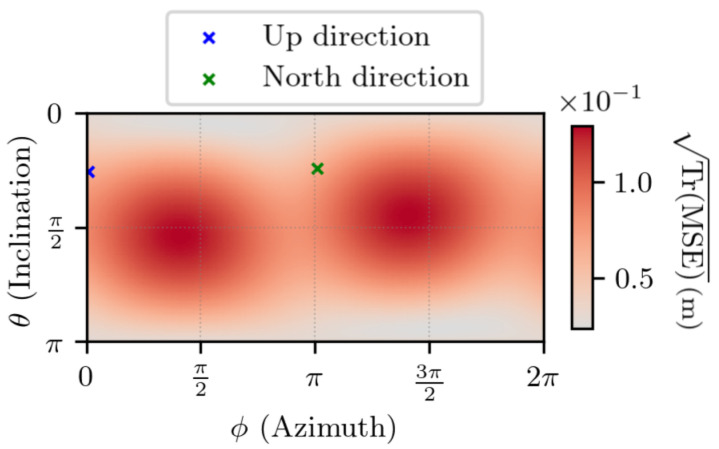
$\sqrt{\mathrm{Tr}\left(\mathrm{MSE}\right)}$ for noiseless measurements, with $∥\Delta r_{1}∥\;=1\;km$, $t_{2}-t_{1}=1\;s$. θ and ϕ are the spherical coordinates, in the ECEF frame, of $\Delta r_{1}$.

In Figure 8 and Figure 9, the set of values taken by $\Delta r_{1}$ corresponds to two spheres with different radii, i.e., for two values of $∥\Delta r_{1}∥$. The time interval is set to $t_{2}-t_{1}=1\;s$. These two plots have the same shape: two modes on the sphere, whose maximum values are close to antipodal and no direction where the estimation error is null. Although these figures look similar, they differ by the magnitude of the values. The local Up and North directions are displayed, but they do not show any link with the errors. More generally, experiments lead to the conclusion that for any value of $∥\Delta r_{1}∥$, the obtained plot has a shape similar to the ones in Figure 8 and Figure 9, and that the set of values taken by $\sqrt{\mathrm{Tr}\left(\mathrm{MSE}\right)}$ is linear with respect to $∥\Delta r_{1}∥$.

In order to show these conclusions, the estimation error is studied in a given direction, which is defined by the angles $\theta =\frac{\pi }{2}$ and $\phi =\frac{\pi }{2}$ in the ECEF frame. The reason why this direction is chosen is that it corresponds approximately to the direction where $\sqrt{\mathrm{Tr}\left(\mathrm{MSE}\right)}$ is maximum for a given value of $∥\Delta r_{1}∥$.

**Figure 10:**
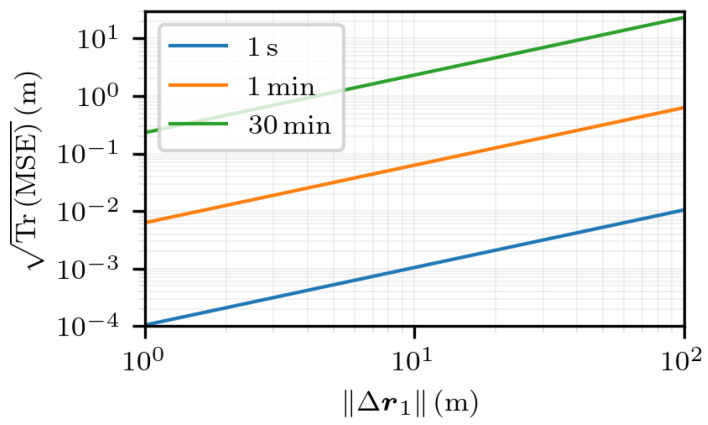
$\sqrt{\mathrm{Tr}\left(\mathrm{MSE}\right)}$ for noiseless measurements and for multiple values of $t_{2}-t_{1}$ as a function of $∥\Delta r_{1}∥$, with $\Delta r_{1}$ in the direction given by the angles $\theta =\frac{\pi }{2}$ and $\phi =\frac{\pi }{2}$ in the ECEF frame.

First, in Figure 10, the estimation error is displayed as a function of $∥\Delta r_{1}∥$ for multiple time intervals. It shows a linear relation between the estimation error and the norm of the first-fix error for all the time intervals, as the slopes of the curves are all equal to 1. The linear coefficient between $\sqrt{\mathrm{Tr}\left(\mathrm{MSE}\right)}$ and $∥\Delta r_{1}∥$ can be read for $∥\Delta r_{1}∥=1\;m$: the greater the time interval, the greater the coefficient. Again, the linearity is hard to predict, as writing the value of $\sqrt{\mathrm{Tr}\left(\mathrm{MSE}\right)}$ as a linear function of $\Delta r_{1}$, when restricted to a specific direction, is not straightforward.

**Figure 11:**
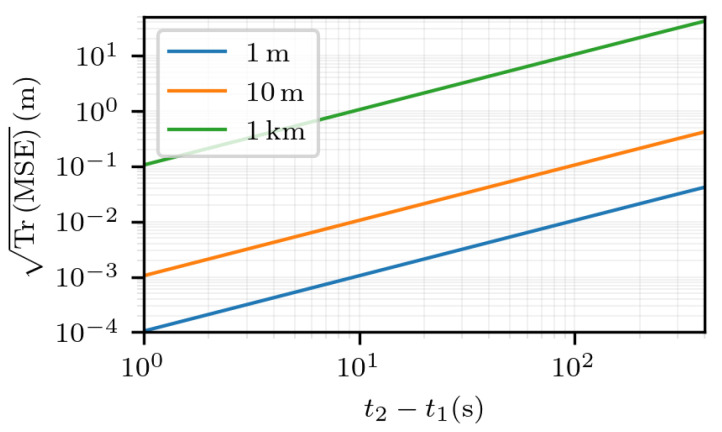
$\sqrt{\mathrm{Tr}\left(\mathrm{MSE}\right)}$ for noiseless measurements and for multiple values of $∥\Delta r_{1}∥$, as a function of $t_{2}-t_{1}$, with $\Delta r_{1}$ in the direction given by the angles $\theta =\frac{\pi }{2}$ and $\phi =\frac{\pi }{2}$ in the ECEF frame.

In Figure 11, the estimation error is displayed as a function of $t_{2}-t_{1}$, for multiple values of $∥\Delta r_{1}∥$. Again, it shows a linear relation between the estimation error $\sqrt{\mathrm{Tr}\left(\mathrm{MSE}\right)}$ and the time interval $t_{2}-t_{1}$, for all the values of $∥\Delta r_{1}∥$. The linear coefficient between $\sqrt{\mathrm{Tr}\left(\mathrm{MSE}\right)}$ and $t_{2}-t_{1}$ can be read for $t_{2}-t_{1}=1\;s$: the greater is $∥\Delta r_{1}∥$, the greater is the coefficient. This result is hard to predict, as $\sqrt{\mathrm{Tr}\left(\mathrm{MSE}\right)}$ is not an explicit function of $t_{2}-t_{1}$.

Finally, Table 3 and Table 4 present visible minimum and maximum values of $\sqrt{\mathrm{Tr}\left(\mathrm{MSE}\right)}$, respectively, for multiple values of $t_{2}-t_{1}$ and for multiple values of $∥\Delta r_{1}∥$. Decomposing $\sqrt{\mathrm{Tr}\left(\mathrm{MSE}\right)}$ in its horizontal and vertical components, in Table 5 and Table 6 are the maximum value of $\sqrt{\mathrm{Tr}\left(\mathrm{MSE}\right)}$ in the horizontal plane and vertical line, respectively, for multiple values of $t_{2}-t_{1}$ and for multiple values of $∥\Delta r_{1}∥$. Omitting rounding errors and cases where both $∥\Delta r_{1}∥$ and $t_{2}-t_{1}$ are very large, both the minimum and maximum values are linear with respect to $∥\Delta r_{1}∥$, but not linear with respect to $t_{2}-t_{1}$. The non-linearity with respect to time is due to the disappearance of G14 after a few minutes, meaning it is not considered in the solution for $t_{2}-t_{1}=30\min$. Again, the linearity is hard to predict, as writing the maximum or minimum values of $\sqrt{\mathrm{Tr}\left(\mathrm{MSE}\right)}$ on a sphere, as a linear function of $∥\Delta r_{1}∥$ is not straightforward. From these tables, conclusions can be made regarding the validity of the first-fix approximation. For example, if one has to estimate a receiver displacement such that $∥\hat{r_{1,2}}-r_{1,2}∥\leq 1.3\;mm$, for a time interval of 1 s, for an initial error of the worst amplitude $∥\Delta r_{1}∥=10\;m$, then the approximation $r_{1}\approx {\hat{r}}_{1}$ is not acceptable, without considering any other source of perturbation.

Overall, it is experimentally shown that the greater $∥\Delta r_{1}∥$, the greater $\sqrt{\mathrm{Tr}\left(\mathrm{MSE}\right)}$, with a linear factor between these two quantities that depends on the time interval $t_{2}-t_{1}$.

### 5.3. Experimental Uncertainty Quantification of Estimated Displacement with Noisy Measurements

In the same scenario as the previous section, noisy measurements are generated following Equation (3), with $\sigma_{\mathrm{TDCP}}=\sqrt{2}\sigma_{\phi }$ and $\sigma_{\phi }=\lambda_{L1}\times 10^{-1}\;m$, with $\lambda_{L1}$ denoting the wavelength of the L1 frequency band. The time interval is set to $t_{2}-t_{1}=1\;s$. Note that alternative noise models could also be considered in order to reflect more realistic weighting schemes. For instance, by incorporating satellite elevation-dependent variances.

As explained previously, since the interest is in the estimation of the receiver displacement vector $\hat{r_{1,2}}$, the quantity $\sqrt{\mathrm{Tr}\left(\mathrm{MSE}\right)}$ is computed by extracting the geometrical part of the MSE matrix. The same process is applied to the MCRB matrix, and the square root of the trace is computed. This choice is justified by the fact that the Loewner partial order has the following property: for all pairs of real symmetric positive semi-definite matrices A and B, $A\geq B\Rightarrow \sqrt{\mathrm{Tr}\left(A\right)}\geq \sqrt{\mathrm{Tr}\left(B\right)}$ (in particular, $\sqrt{\mathrm{Tr}\left(A\right)}\geq 0$).

**Figure 12:**
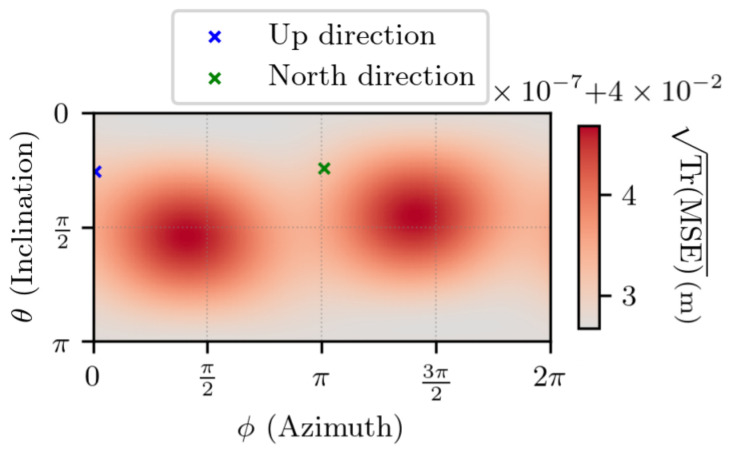
$\sqrt{\mathrm{Tr}\left(\mathrm{MSE}\right)}$ for noisy measurements, with $∥\Delta r_{1}∥\;=1\;m$, $t_{2}-t_{1}=1\;s$. θ and ϕ are the spherical coordinates in the ECEF frame of $\Delta r_{1}$.

**Figure 13:**
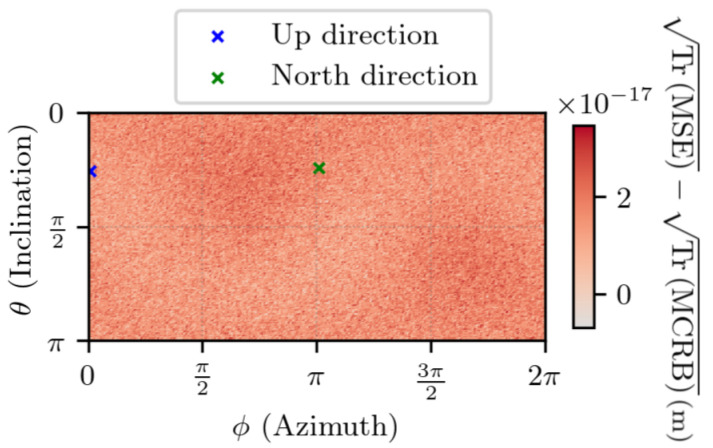
Difference between $\sqrt{\mathrm{Tr}\left(\mathrm{MSE}\right)}$ and $\sqrt{\mathrm{Tr}\left(\mathrm{MCRB}\right)}$ for noisy measurements with $∥\Delta r_{1}∥\;=1\;m$, $t_{2}-t_{1}=1\;s$. θ and ϕ are the spherical coordinates in the ECEF frame of $\Delta r_{1}$. The negative values exist due to numerical errors.

Figure 12 shows $\sqrt{\mathrm{Tr}\left(\mathrm{MSE}\right)}$ for $∥\Delta r_{1}∥\;=1\;m$. On the same set, the difference between $\sqrt{\mathrm{Tr}\left(\mathrm{MSE}\right)}$ and its lower bound $\sqrt{\mathrm{Tr}\left(\mathrm{MCRB}\right)}$ is visible in Figure 13. The results indicate that, as was the case in the absence of noise, the error is characterized by two modes on the sphere. Also, the estimator’s error is close to the lowest value that it can reach, with no particular direction appearing.

**Figure 14:**
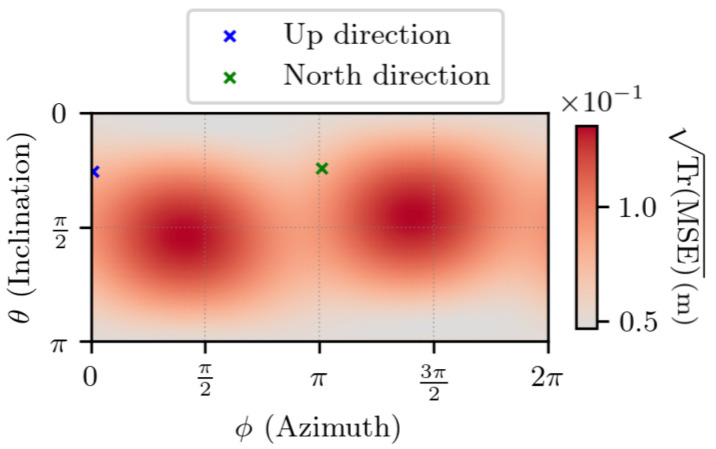
$\sqrt{\mathrm{Tr}\left(\mathrm{MSE}\right)}$ for noisy measurements, with $∥\Delta r_{1}∥\;=1\;km$, $t_{2}-t_{1}=1\;s$. θ and ϕ are the spherical coordinates in the ECEF frame of $\Delta r_{1}$.

**Figure 15:**
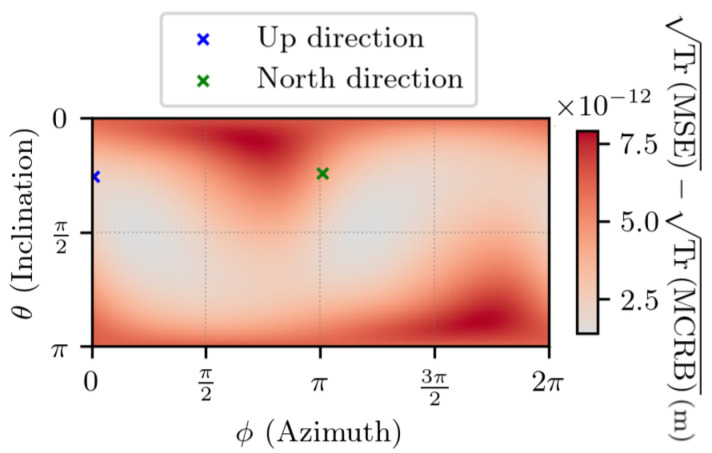
Difference between $\sqrt{\mathrm{Tr}\left(\mathrm{MSE}\right)}$ and $\sqrt{\mathrm{Tr}\left(\mathrm{MCRB}\right)}$ for noisy measurements with $∥\Delta r_{1}∥\;=1\;km$, $t_{2}-t_{1}=1\;s$. θ and ϕ are the spherical coordinates in the ECEF frame of $\Delta r_{1}$.

Figure 14 and Figure 15 depict the same experiment, with $∥\Delta r_{1}∥\;=1\;km$. Again, the results indicate that the estimator’s error is close to the lowest value that it can reach.

**Figure 16:**
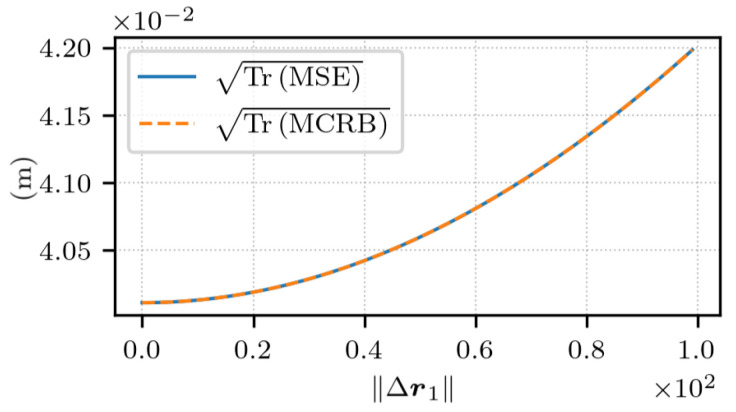
$\sqrt{\mathrm{Tr}\left(\mathrm{MSE}\right)}$ and $\sqrt{\mathrm{Tr}\left(\mathrm{MCRB}\right)}$ for noisy measurements as a function of $∥\Delta r_{1}∥$, with $t_{2}-t_{1}=1\;s$ and $\Delta r_{1}$ in the direction given by the angles $\theta =\frac{\pi }{2}$ and $\phi =\frac{\pi }{2}$ in the ECEF frame.

Finally, as done previously, the estimation error is studied along an axis defined by the angles $\theta =\frac{\pi }{2}$ and $\phi =\frac{\pi }{2}$ in the ECEF frame. Figure 16 shows the error and its lower bound. At $\Delta r_{1}=0$, the estimation error is due to the variance of the additive noise within the observations. This noise term adds up to the bias as $∥\Delta r_{1}∥$ increases, which produces the curve that is observed. For large values of $∥\Delta r_{1}∥$, the error is mainly due to the misspecified model. Also, the results indicate that the estimator’s error has reached the lowest value possible.

**Figure 17:**
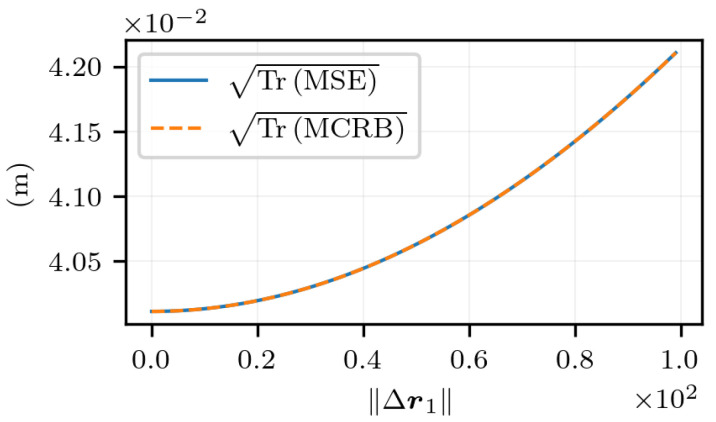
Maximum value of $\sqrt{\mathrm{Tr}\left(\mathrm{MSE}\right)}$ and corresponding $\sqrt{\mathrm{Tr}\left(\mathrm{MCRB}\right)}$ for noisy measurements as a function of $∥\Delta r_{1}∥$ with $t_{2}-t_{1}=1\;s$.

In the last experiment, Figure 17 depicts the maximum value of $\sqrt{\mathrm{Tr}\left(\mathrm{MSE}\right)}$ as a function of $∥\Delta r_{1}∥$.

To conclude this section, one can say that all the estimators built in these experiments are close to the MCRB. The mean error depends on the amount of noise in the observations, and on the misspecification of the model used.

## 6. Conclusions and Future Work

The TDCP model is expressed as a function of a GNSS receiver displacement and clock bias difference. The impact on the estimated displacement of the uncertainty regarding the receiver coordinates at the first epoch has been quantified for a given geometry at a given location. A similar study can be run in real-time by any user anywhere in the world, as the proposed method does not rely on final products, and real-time ephemerides would provide similar results. Considering the displacement estimation error due to a misspecified first fix only from a set of TDCP observations and given criteria on the precision of the estimated displacement, the provided tables are an indicator of the validity of the first fix. It is experimentally shown that for long time intervals, the impact is important. This is due to the fact that for long time intervals, the satellite coordinates change more, and lead to different receiver-to-satellite unit vectors. This effect would be more prominent when the considered satellites are at a low altitude. Therefore, when considering Low Earth Orbit satellites, whose displacements are much faster than GNSS satellites in Medium Earth Orbits, the impact would be greater. One might also notice that the same experiment could be transposed to an RTK framework: at a given epoch, the station coordinates are known, the baseline vector corresponds to the receiver displacement, and the single difference between receivers would correspond to TDCPs.

## Figures and Tables

**Table 1:** Link between the terms introduced in Section 3 and Section 4.

Estimation	TDCP
θ as in Equation (9)	${\left(r_{1,2}^{\;T},dt_{1,2}^{r}\right)}^{\;T}$ as in Equation (4)
y as in Equation (9)	$\lambda {\left({\left({\tilde{\Phi }}_{2}-{\tilde{\Phi }}_{1}\right)}^{1},\ldots ,{\left({\tilde{\Phi }}_{2}-{\tilde{\Phi }}_{1}\right)}^{m}\right)}^{\;T}$
	with $\lambda {\left({\tilde{\Phi }}_{2}-{\tilde{\Phi }}_{1}\right)}^{i}$ as in Equation (3)
*g* as in Equation (9)	$\left(f_{r_{1}}^{1},\ldots ,f_{r_{1}}^{m}\right)$, with $f_{r_{1}}^{i}$ as in Equation (5)
*h* as in Equation (10)	$\left(f_{\hat{r_{1}}}^{1},\ldots ,f_{\hat{r_{1}}}^{m}\right)$, with $f_{\hat{r_{1}}}^{i}$ as in Equation (6)
R as in Equation (9)	$\mathrm{diag}\left(\sigma_{{\mathrm{TDCP}}_{1}}^{2},\ldots ,\sigma_{{\mathrm{TDCP}}_{m}}^{2}\right)$
	where $\sigma_{{\mathrm{TDCP}}_{i}}^{2}$ is the variance of ε Equation (3)

**Table 2:** Maximum value of Δf Equation (7) with respect to $∥\Delta r_{1}∥$ and $t_{2}-t_{1}$.

	$t_{2}-t_{1}$	1 s	1 min	30 min
$∥\Delta r_{1}∥$				
1 m		1.2 × 10^−4^ m	7.1 × 10^−3^ m	2.1 × 10^−1^ m
10 m		1.2 × 10^−3^ m	7.1 × 10^−2^ m	2.1 m
1 km		1.2 × 10^−1^ m	7.1 m	2.1 × 10^1^ m
1000 km		1.2 × 10^2^ m	7.1 × 10^3^ m	2.1 × 10^5^ m

**Table 3:** Min values of $\sqrt{\mathrm{Tr}\left(\mathrm{MSE}\right)}$ with respect to $∥\Delta r_{1}∥$ and $t_{2}-t_{1}$.

	$t_{2}-t_{1}$	1 s	1 min	30 min
$∥\Delta r_{1}∥$				
1 m		2.4 × 10^−5^ m	1.3 × 10^−3^ m	7.7 × 10^−2^ m
10 m		2.4 × 10^−4^ m	1.3 × 10^−2^ m	7.7 × 10^−1^ m
1 km		2.4 × 10^−2^ m	1.3 m	7.7 × 10^1^ m
1000 km		2.4 × 10^1^ m	1.3 × 10^3^ m	7 × 10^4^ m

**Table 4:** Max values of $\sqrt{\mathrm{Tr}\left(\mathrm{MSE}\right)}$ with respect to $∥\Delta r_{1}∥$ and $t_{2}-t_{1}$.

	$t_{2}-t_{1}$	1 s	1 min	30 min
$∥\Delta r_{1}∥$				
1 m		1.3 × 10^−4^ m	7.7 × 10^−3^ m	3.8 × 10^−1^ m
10 m		1.3 × 10^−3^ m	7.7 × 10^−2^ m	3.8 m
1 km		1.3 × 10^−1^ m	7.7 m	3.8 × 10^2^ m
1000 km		1.3 × 10^2^ m	7.8 × 10^3^ m	3.8 × 10^5^ m

**Table 5:** Max values of $\sqrt{\mathrm{Tr}\left(\mathrm{MSE}\right)}$ in the receiver local horizontal plane with respect to $∥\Delta r_{1}∥$ and $t_{2}-t_{1}$.

	$t_{2}-t_{1}$	1 s	1 min	30 min
$∥\Delta r_{1}∥$				
1 m		8.8 × 10^−5^ m	5.3 × 10^−3^ m	1.7 × 10^−1^ m
10 m		8.8 × 10^−4^ m	5.3 × 10^−2^ m	1.7 m
1 km		8.8 × 10^−2^ m	5.3 m	1.7 × 10^2^ m
1000 km		8.9 × 10^1^ m	5.3 × 10^3^ m	1.7 × 10^5^ m

**Table 6:** Max values of $\sqrt{\mathrm{Tr}\left(\mathrm{MSE}\right)}$ on the receiver local vertical line with respect to $∥\Delta r_{1}∥$ and $t_{2}-t_{1}$.

	$t_{2}-t_{1}$	1 s	1 min	30 min
$∥\Delta r_{1}∥$				
1 m		1.1 × 10^−4^ m	6.7 × 10^−3^ m	3.2 × 10^−1^ m
10 m		1.1 × 10^−3^ m	6.7 × 10^−2^ m	3.2 m
1 km		1.1 × 10^−1^ m	6.7 m	3.2 × 10^2^ m
1000 km		1.2 × 10^2^ m	6.7 × 10^3^ m	3.2 × 10^5^ m

## Data Availability

The original contributions presented in the study are included in the article, further inquiries can be directed to the corresponding author.

## Appendix A. Direction of Null and Extremum Model Error

Recalling that $\Delta r_{1}={\hat{r}}_{1}-r_{1}$ and that Equation (8) is the function Δf that maps an error $\Delta r_{1}$ to a model error:

$$\begin{array}{cc} \Delta f(\Delta r_{1}) & =∥s_{2}-r_{1}-r_{1,2}-\Delta r_{1}∥\\ \; & -∥s_{2}-r_{1}-r_{1,2}∥\\ \; & -(∥s_{1}-r_{1}-\Delta r_{1}∥-∥s_{1}-r_{1}∥) \end{array}$$

where $∥x∥=\sqrt{\langle x,x\rangle }$ and $\langle x,y\rangle =x^{\;T}y$ is the dot product on $R^{3}$. Defining $a≜s_{2}-r_{1}-r_{1,2}$ and $b≜s_{1}-r_{1}$, one obtains

$$\Delta f\left(\Delta r_{1}\right)=∥a-\Delta r_{1}∥-∥a∥-\left(∥b-\Delta r_{1}∥-∥b∥\right).$$

Given that Δf(0)=0, one obtains

$$\begin{array}{cc} \Delta f(\Delta r_{1}) & =d(\Delta f{)}_{0}\left(\Delta r_{1}\right)+o\left(∥\Delta r_{1}∥\right)\\ \; & =-\langle \frac{a}{∥a∥}-\frac{b}{∥b∥},\Delta r_{1}\rangle +o\left(∥\Delta r_{1}∥\right) \end{array}$$

where $d(\Delta f{)}_{0}\left(\Delta r_{1}\right)$ is the differential of Δf at 0, evaluated at $\Delta r_{1}$. If this term had to be expressed in the canonical bases of $R^{3}$ and R, it would be equal to the Jacobian matrix of Δf at 0 multiplied by $\Delta r_{1}$. Therefore, in a neighborhood of 0, the linear approximation of Δf is given by:

$$\Delta f\left(\Delta r_{1}\right)\approx -\langle \frac{a}{∥a∥}-\frac{b}{∥b∥},\Delta r_{1}\rangle .$$

Now, one has to study the vector $\frac{a}{∥a∥}-\frac{b}{∥b∥}$ and give it a geometrical interpretation, other than being the difference between two very close unit vectors. One defines the function:

$$\gamma :R^{3}\to R^{3},x\mapsto \frac{a}{∥a∥}-\frac{a+x}{∥a+x∥},$$

and defining $v≜b-a=-\left(s_{2}-s_{1}-r_{1,2}\right)$, one obtains

$$\frac{a}{∥a∥}-\frac{b}{∥b∥}=\frac{a}{∥a∥}-\frac{a+v}{∥a+v∥}=\gamma \left(v\right).$$

Now that the vector $\frac{a}{∥a∥}-\frac{b}{∥b∥}$ is expressed as a valuation of the function γ, a linear approximation of it is given: it can be shown that $\forall \alpha \in R^{3}\setminus \left\{-a\right\},\forall x\in R^{3}$:

$$\gamma \left(\alpha +x\right)=\gamma \left(\alpha \right)+d\gamma_{\alpha }\left(x\right)+o\left(∥x∥\right)$$

where $d\gamma_{\alpha }\left(x\right)$ is the differential of γ at α, evaluated at x. If this term had to be expressed in the canonical basis of $R^{3}$, it would be equal to the Jacobian matrix of γ at α multiplied by x.

One can show that:

$$d\gamma_{\alpha }\left(x\right)=-\frac{1}{∥a+\alpha ∥}\left(x-\frac{a+\alpha }{∥a+\alpha ∥}\langle \frac{a+\alpha }{∥a+\alpha ∥},x\rangle \right).$$

Since γ(0)=0, the linear approximation of the function γ around 0 is given by:

$$\gamma \left(x\right)\approx -\frac{1}{∥a∥}\left(x-\frac{a}{∥a∥}\langle \frac{a}{∥a∥},x\rangle \right).$$

As the vector v is an infinitesimal and orthogonal vector, with respect to the vector $a=s_{2}-r_{2}$, the scalar product term $\langle \frac{a}{∥a∥},v\rangle$ is neglected:

$$\Rightarrow \frac{a}{∥a∥}-\frac{b}{∥b∥}=\gamma \left(v\right)\approx -\frac{v}{∥a∥}=\frac{a-b}{∥a∥}.$$

Finally, $\Delta f\left(\Delta r_{1}\right)\approx 0\Leftrightarrow \langle \frac{a-b}{∥a∥},\Delta r_{1}\rangle \approx 0$, i.e., the model error is null for any error $\Delta r_{1}$ orthogonal to $a-b=s_{2}-s_{1}-r_{1,2}$, and the directions of extremum error are given by the vector $s_{2}-s_{1}-r_{1,2}$.

## Appendix B. Model Error Along the Worst Direction

As a reminder, in Appendix A, the vectors $a≜s_{2}-r_{1}-r_{1,2}$ and $b≜s_{1}-r_{1}$ are defined, and the vector v=a−b is shown to be the vector that defines the direction of extremum error. Here, the model error Δf of Equation (7) is studied along a one-dimensional subspace: for any unit vector e, considering the application:

$$\begin{array}{cc} \phi :R & \to R\\ \lambda & \mapsto \Delta f\left(\lambda e\right) \end{array}$$

with

$$\begin{array}{cc} \Delta f\left(\lambda e\right) & =∥a-\lambda e∥-∥a∥\\ \; & -\left(∥b-\lambda e∥-∥b∥\right), \end{array}$$

one finds out that the derivative of ϕ at λ is given by:

$$\phi^{\prime }\left(\lambda \right)=-\frac{\langle a-\lambda e,e\rangle }{∥a-\lambda e∥}+\frac{\langle b-\lambda e,e\rangle }{∥b-\lambda e∥}.$$

Finally, if $e=\frac{v}{∥v∥}$ and $\Delta r_{1}=\lambda e,\lambda \in R$, then $∥\Delta r_{1}∥=\left|\lambda \right|$ and one has obtained the derivative of ϕ at $∥\Delta r_{1}∥$ along the direction of v.
